# New Coleoptera records from New Brunswick, Canada: Eucnemidae

**DOI:** 10.3897/zookeys.179.2492

**Published:** 2012-04-04

**Authors:** Reginald P. Webster, Jon D. Sweeney, Ian DeMerchant

**Affiliations:** 1 Natural Resources Canada, Canadian Forest Service - Atlantic Forestry Centre, 1350 Regent St., P.O. Box 4000, Fredericton, NB, Canada E3B 5P7

**Keywords:** Eucnemidae, taxonomy, Canada, New Brunswick, new records, Lindgren funnel traps

## Abstract

We report nine species of Eucnemidae new to the province and additional records for *Onichodon canadensis* (Brown) and *Dromaeolus harringtoni* Horn. Five species, *Xylophilus cylindriformis* (Horn), *Entomophthalmus rufiolus* (LeConte), *Stethon pectorosus* LeConte, *Onichodon orchesides* Newman, and *Isarthrus rufipes* (Melsheimer), are newly recorded for the Maritime provinces. This brings the total number of Eucnemidae recorded from New Brunswick to 15 species. Lindgren funnel traps are an effective tool for sampling the Eucnemidae.

## Introduction

The Nearctic Eucnemidae (false click beetles) was revised by Muona (2000), and this treatment is indispensible for identifying members of this family from Canada. Eucnemidae larvae develop in wood infected by fungi that cause white rot, but details of the biology of most species are unknown (Muona 2000). The Eucnemidae appear to be good indicators of diverse forest structure, and the decline in populations of species in this family may be associated with forest management practices that promote a loss of deadwood and coarse woody debris (Muona 2000). Majka (2007), in a review of the Eucnemidae of the Maritime provinces (New Brunswick, Nova Scotia, Prince Edward Island) of Canada, discussed the apparent rarity of members of this family in the context of the forest practices in the region and the role these practices may have on population numbers.

Majka (2007) reported six species of Eucnemidae from New Brunswick. *Onichodon canadensis* (Brown) was reported as new. Intensive collecting of Coleoptera in New Brunswick by the first author and records obtained from by-catch samples during a study to develop a general attractant for the detection of invasive species of Cerambycidae have yielded additional provincial records. The goals of this contribution are to publish these new records and to report the apparent utility of Lindgren funnel traps (Lindgren 1983) for collecting Eucnemidae specimens. We also report additional provincial specimen records for two species hitherto represented in New Brunswick by only one specimen, and collection methods for all the Eucnemidae currently known from the province.

## Methods and conventions

The following records are based on specimens collected as part of a general survey by the first author to document the Coleoptera fauna of New Brunswick. Additional provincial records were obtained from specimens contained in the collection at the Atlantic Forestry Centre, Natural Resources Canada, Canadian Forest Service, Fredericton, New Brunswick.

### Collection methods

Various collection methods were employed to collect the Eucnemidae reported in this study. Details are outlined in Webster et al. (2009, Appendix). Many specimens were collected as by-catch in Lindgren 12-funnel traps (ConTech Inc., Delta, BC) baited with various attractants as part of a study to develop a general attractant for the detection of invasive species of Cerambycidae. These traps mimic tree trunks and are often effective for sampling species of Coleoptera that live in microhabitats associated with standing trees (Lindgren 1983). Details on the methods used for deployment of these traps are outlined in Webster et al. (in press). A description of the habitat was recorded for all specimens collected during this survey. Locality and habitat data are presented exactly as on labels for each record. This information, as well as additional collecting notes, are summarized in the collection and habitat data section for each species.

### Distribution

Distribution maps, created using ArcMap and ArcGIS, are presented for each species in New Brunswick. Every species is cited with current distribution in Canada and Alaska, using abbreviations for the state, provinces, and territories. New records for New Brunswick are indicated in bold under Distribution in Canada and Alaska. The following abbreviations are used in the text:

**Table:** 

**AK**	Alaska	**MB**	Manitoba
**YT**	Yukon Territory	**ON**	Ontario
**NT**	Northwest Territories	**QC**	Quebec
**NU**	Nunavut	**NB**	New Brunswick
**BC**	British Columbia	**PE**	Prince Edward Island
**AB**	Alberta	**NS**	Nova Scotia
**SK**	Saskatchewan	**NF & LB**	Newfoundland and Labrador

Acronyms of collections examined or where specimens reside referred to in this study are as follows:

**AFC** Atlantic Forestry Centre, Natural Resources Canada, Canadian Forest Service, Fredericton, New Brunswick, Canada

**CNC** Canadian National Collection of Insects, Arachnids and Nematodes, Agriculture and Agri-Food Canada, Ottawa, Ontario, Canada

**NBM** New Brunswick Museum, Saint John, New Brunswick, Canada

**RWC** Reginald P. Webster Collection, Charters Settlement, New Brunswick, Canada

## Results

We report nine species new to the province of New Brunswick and additional records for *Onichodon canadensis* (Brown) and *Dromaeolus harringtoni* Horn. Five species, *Xylophilus cylindriformis* (Horn), *Entomophthalmus rufiolus* (LeConte), *Stethon pectorosus* LeConte, *Onichodon orchesides* Newman, and *Isarthrus rufipes* (Melsheimer), are newly recorded for the Maritime provinces. This brings the total number of Eucnemidae recorded from New Brunswick to 15 species (Table 1).

**Table 1.** Eucnemidae recorded from New Brunswick, Canada and number collected using various collection methods

**Species**	**Lindgren funnel traps**	**Window traps**	**Other collecting methods^1^**
**Subfamily Melasinae Fleming**			
**Tribe Melasini Fleming**			
*Isorhipis obliqua* (Say)	26	4	7
**Tribe Xylobiini Reitter**			
*Xylophilus cylindriformis* (Horn)**	13		
**Tribe Epiphanini Muona**			
*Epiphanis cornutus* (Eschscholtz)	30		1
*Hylis terminalis* (LeConte)*	21	1	1
**Tribe Dirhagini Reitter**			
*Microrhagus pectinatus* LeConte	4	1	1
*Microrhagus subsinuatus* LeConte*	22		1
*Microrhagus triangularis* (Say)*	6		1
*Entomophthalmus rufiolus* (LeConte)**	4		
**Subfamily Eucneminae Eschscholtz**			
**Tribe Mesogenini Muona**			
*Stethon pectorosus* LeConte**	1		
**Subfamily Macraulacinae Fleutiaux**			
**Tribe Macraulacini Fleutiaux**			
*Onichodon canadensis* (Brown)	37	1	2
*Onichodon orchesides* Newman**	6		
*Isarthrus rufipes* (Melsheimer)**	2		
*Dromaeolus harringtoni* Horn	28		1
*Deltometopus amoenicornis* (Say)	10		5
**Tribe Nematodini Leiler**			
*Nematodes penetrans* (LeConte)*	28		
**Total**	238	7	20

**Notes:** *New to province; **New to Maritime provinces. ^1^ Other collecting methods include hand collecting, sweeping, and beating foliage.

### Species Accounts

All records below are species newly recorded for New Brunswick, Canada, unless noted otherwise (additional records). Species followed by ** are newly recorded from the Maritime provinces of Canada.

The classification of the Eucnemidae follows Muona (1993).

### Family Eucnemidae Eschscholtz, 1829

**Subfamily Melasinae Fleming, 1821**

**Tribe Xylobiini Reitter, 1911**

#### Xylophilus cylindriformis

(Horn, 1871)**

http://species-id.net/wiki/Xylophilus_cylindriformis

Map 1

##### Material examined.

**New Brunswick, Carleton Co.**, Jackson Falls, “Bell Forest”, 46.2200°N, 67.7231°W, 12–19.VI.2008, 5–12.VII.2008, 12–19.VII.2008, R. P. Webster, mature hardwood forest, Lindgren funnel traps (7, AFC, RWC); same locality and habitat but 28.VI–7.VII.2009, 7–14.VII.2009, 19–31.VII.2009, R. Webster & M.-A. Giguère, Lindgren funnel traps (6, AFC, RWC).

**Map 1.**
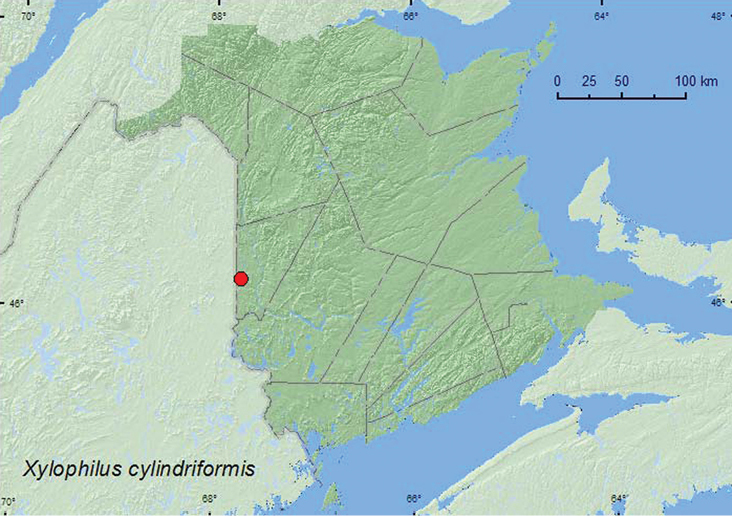
Collection localities in New Brunswick, Canada of *Xylophilus cylindriformis*.

##### Collection and habitat data.

Muona (2000) reported adults from a window trap and Malaise trap, otherwise little is known about the biology of this species. Levesque and Levesque (1993) collected a specimen in Québec at the boundary between a raspberry (*Rubus idaeus* L.) plantation and a white pine (*Pinus strobus* L.) woodland. Adults from New Brunswick were captured in Lindgren funnel traps deployed in a mature hardwood forest with American beech (*Fagus grandifolia* Ehrh.), sugar maple (*Acer saccharum* Marsh), and ash (*Fraxinu*s sp.). Adults were captured during June and July.

##### Distribution in Canada and Alaska.

BC, ON, QC, **NB** (Bousquet 1991; Muona 2000). Muona (2000) reported this species from California east to Wisconsin and New Hampshire in the United States.

### Tribe Epiphanini Muona, 1993

#### Hylis terminalis

(LeConte, 1866)

http://species-id.net/wiki/Hylis_terminalis

Map 2

##### Material examined.

**New Brunswick, Carleton Co.**, Jackson Falls, Bell Forest, 46.2200°N, 67.7231°W, 12–19.VII.2008, R. P. Webster, mature hardwood forest, Lindgren funnel trap (1, AFC); same locality and habitat but 7–12.VIII.2009, R. Webster & M.-A. Giguère, Lindgren funnel trap (1, RWC). **Charlotte Co.**, 10 km NW of New River Beach, 45.2110°N, 66.6170°W, 10–23.VIII.2010, C. Hughes & K. Burgess, old growth eastern white cedar forest, Lindgren funnel trap (1, NBM). **Northumberland Co.**, Priceville, 27.VII.1972, N. Carter, window trap (1, AFC). **Queens Co.**, Cranberry Lake P.N.A. (Protected Natural Area), 46.1125°N, 65.6075°W, 14–19.VIII.2009, R. Webster & M.-A. Giguère, old red oak forest, Lindgren funnel trap (1, AFC); same locality data and forest type, 7–13.VII.2011, 13–20.VII.2011, 20.VII–4.VIII.2011, 4–18.VIII.2011, 18–31.VIII.2011, M. Roy & V. Webster, Lindgren funnel traps in forest canopy (12, AFC, NBM, RWC); Grand Lake Meadows P.N.A., 45.8227°N, 66.1209°W, 29.VI-12.VII.2010, R. Webster, C. MacKay, M. Laity, & R. Johns, old silver maple forest with green ash and seasonally flooded marsh, Lindgren funnel trap (1, RWC). **Sunbury Co.**, Acadia Research Forest, 45.9866°N, 66.3841°W, 13–21.VII.2009, 21–29.VII.2009, R. Webster & M.-A. Giguère, red spruce forest with red maple and balsam fir, Lindgren funnel traps (2, RWC). **York Co.**, Charters Settlement, 45.8430°N, 66.7275°W, 11.VII.2005, R. P. Webster, regenerating mixed forest, beating foliage (1, RWC); 15 km W of Tracy off Rt. 645, 45.6848°N, 66.8821°W, 14–20.VII.2009, 20–29.VII.2009, R. Webster & M.-A. Giguère, old red pine forest, Lindgren funnel traps (2, AFC, RWC); same locality and habitat data but 30.VI–13.VII.2009, R. Webster, K. Burgess, & C. Hughes, Lindgren funnel traps (3, AFC, RWC).

**Map 2.**
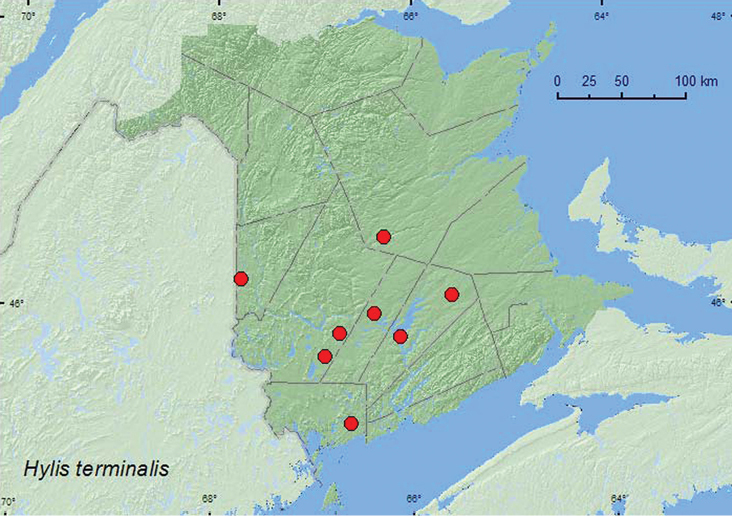
Collection localities in New Brunswick, Canada of *Hylis terminalis*.

##### Collection and habitat data.

*Hylis terminalis* has been reared from *Carya* (Horn 1886) and a moist, decayed American beech log (Knull 1946). This specieswas found in various forest types in New Brunswick. These included a mature hardwood forest with beech, sugar maple, and ash, an old red oak (*Quercus rubra* L.) forest, an old silver maple (*Acer saccharinum* L.) forest with green ash (*Fraxinus pennsylvanica* Marsh), a red spruce (*Picea rubens* Sarg.) forest with red maple (*Acer rubrum* L.) and balsam fir (*Abies balsamea* (L.) Mill.), an old-growth eastern white cedar (*Thuja occidentalis* L.) forest, and an old red pine (*Pinus resinosa* Ait.) forest. Most adults were captured in Lindgren funnel traps. One individual was collected by beating foliage in a regenerating mixed forest, and another was caught in a window trap. Adults were captured during July and August.

##### Distribution in Canada and Alaska.

MB, ON, QC, **NB**, PE, NS (Bousquet 1991; Muona 2000; Majka 2007). Records from western Canada (BC, AB, SK) reported in Bousquet (1991) are in error according to Muona (2000).

### Tribe Dirhagini Reitter, 1911

#### Microrhagus subsinuatus

LeConte, 1852

http://species-id.net/wiki/Microrhagus_subsinuatus

Map 3

##### Material examined.

**New Brunswick, Carleton Co.**, Jackson Falls, Bell Forest, 46.2200°N, 67.7231°W, 5–12.VII.2008, 12–19.VII.2008, 19–28.VII.2008, R. P. Webster, mature hardwood forest, Lindgren funnel traps (5, AFC, RWC); same locality and habitat but 7–12.VIII.2009, R. Webster & M.-A. Giguère, Lindgren funnel trap (1, RWC); same locality and habitat but, 13.VIII.2007, R. P. Webster, sweeping foliage (1, RWC). **Charlotte Co.**, 10 km NW of New River Beach, 45.2110°N, 66.6170°W, 29.VI-16.VII.2010, R. Webster & C. MacKay, old growth eastern white cedar forest, Lindgren funnel trap (1, RWC). **Queens Co.**, Grand Lake Meadows P.N.A., 45.8227°N, 66.1209°W, 29.VI–12.VII.2010, 12–26.VII.2010, R. Webster, C. MacKay, M. Laity, & R. Johns, old silver maple forest with green ash and seasonally flooded marsh, Lindgren funnel traps (5, AFC, RWC); same locality data and forest type, 19.VII–5.VIII.2011, M. Roy & V. Webster, Lindgren funnel trap (1, NBM). **Restigouche Co.**, Dionne Brook P.N.A., 47.9030°N, 68.3503°W, 28.VII–9.VIII.2011, M. Roy & V. Webster, old-growth northern hardwood forest, Lindgren funnel trap (1, RWC); same locality and collectors but 47.9064°N, 68.3441°W, 14–28.VII.2011, 28.VII–9.VIII.2011, old-growth white spruce and balsam fir forest, Lindgren funnel traps (6, AFC, NBM, RWC). **Sunbury Co.**, Acadia Research Forest, 45.9866°N, 66.3841°W, 21–29.VII.2009, R. Webster & M.-A. Giguère, red spruce forest with red maple and balsam fir, Lindgren funnel trap (1, RWC).

**Map 3.**
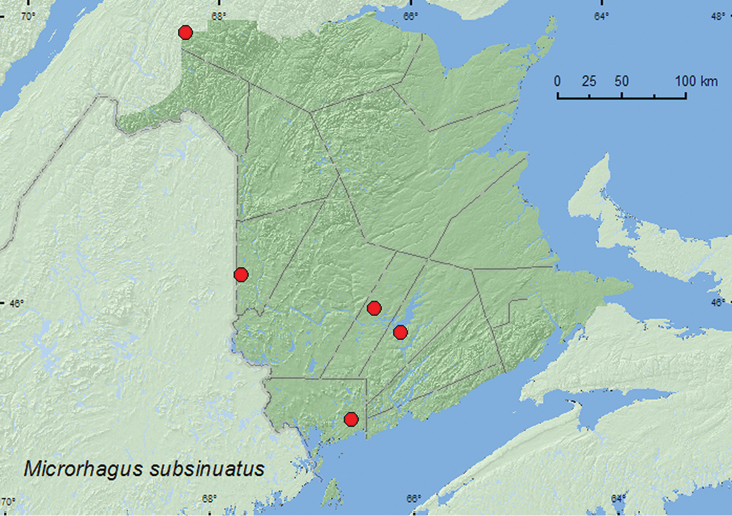
Collection localities in New Brunswick, Canada of *Microrhagus subsinuatus*.

##### Collection and habitat data.

*Microhagus subsinuatus* was reared from a moist, decayed *Fagus grandifolia* log (Knull 1946) and swept from milkweed (Blatchley 1910). This species was found in various forest types in New Brunswick. These included a mature hardwood forest with American beech, sugar maple, and ash, an old silver maple forest with green ash, a red spruce forest with red maple and balsam fir, an old-growth northern hardwood forest, an old-growth white spruce and balsam fir forest, and an old-growth eastern white cedar forest. Most adults were captured in Lindgren funnel traps. One individual was swept from foliage in a mature hardwood forest. Adults were collected during June, July, and August.

##### Distribution in Canada and Alaska.

MB, ON, QC, **NB**, PE, NS (Bousquet 1991; Muona 2000; Majka 2007).

#### Microrhagus triangularis

(Say, 1823)

http://species-id.net/wiki/Microrhagus_triangularis

Map 4

##### Material examined.

**New Brunswick, Carleton Co.**, Jackson Falls, Bell Forest, 46.2200°N, 67.7231°W, 28.VII–6.VIII.2008, R. P. Webster, mature hardwood forest, Lindgren funnel traps (2, RWC). **Charlotte Co.**, 10 km NW of New River Beach, 45.2110°N, 66.6170°W, 29.VI–16.VII.2010, R. Webster & C. MacKay, old growth eastern white cedar forest, Lindgren funnel trap (1, AFC). **York Co.**, Charters Settlement, 45.8430°N, 66.7275°W, 20.VII.2008, R. P. Webster, regenerating mixed forest, beating foliage (1, RWC); 15 km W of Tracy off Rt. 645, 45.6848°N, 66.8821°W, 30.VI–13.VII.2010, 12–27.VII.2010, R. Webster & C. MacKay, old red pine forest, Lindgren funnel traps (3, AFC).

**Map 4.**
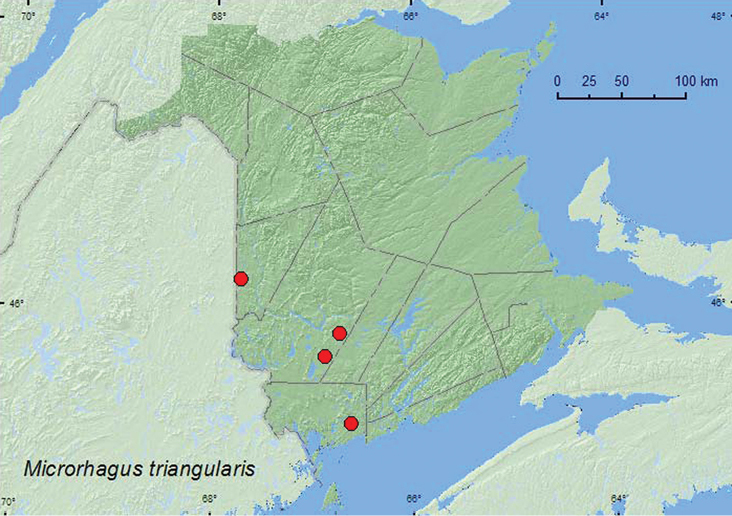
Collection localities in New Brunswick, Canada of *Microrhagus triangularis*.

##### Collection and habitat data.

Adults of *Microrhagus triangularis* were reported from *Cornus* logs, otherwise little is known about the biology and habitat requirements of this species (Muona 2000). Levesque and Levesque (1993) collected two specimens in Québec at the boundary between a raspberry plantation and a white pine woodland. In New Brunswick, adults of this common species were found in a mature hardwood forest with beech, sugar maple, and ash, an old red oak forest, an old-growth eastern white cedar forest, and an old red pine forest. Most adults were captured in Lindgren funnel traps. One individual was collected by beating foliage in a regenerating mixed forest. Adults were collected during July and August.

##### Distribution in Canada and Alaska.

ON, QC, **NB**, NS (Bousquet 1991; Majka 2007).

#### Entomophthalmus rufiolus

(LeConte, 1866)**

http://species-id.net/wiki/Entomophthalmus_rufiolus

Map 5

##### Material examined.

**New Brunswick, Queens Co.**, Grand Lake Meadows P.N.A., 45.8227°N, 66.1209°W, 5–19.VII.2011, 19.VII–5.VIII.2011, M. Roy & V. Webster, old silver maple forest and seasonally flooded marsh, Lindgren funnel traps (2, RWC). **Sunbury Co.**, Burton, near Sunpoke Lake, 45.7658°N, 66.5546°W , 24.VII–1.VIII.2008, R. P. Webster, oak forest with scattered white pine, Lindgren funnel trap (1, RWC); Acadia Research Forest, 45.9866°N, 66.3841°W, 8–13.VII.2009, R. Webster & M.-A. Giguère, red spruce forest with red maple and balsam fir, Lindgren funnel trap (1, RWC).

**Map 5.**
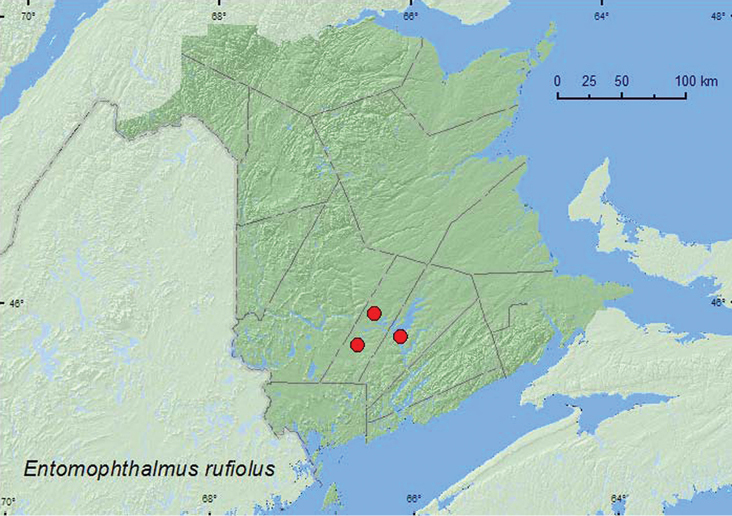
Collection localities in New Brunswick, Canada of *Entomophthalmus rufiolus*.

##### Collection and habitat data.

*Entomophthalmus rufiolus* has been collected from hickory (*Carya* sp.), at black-light traps, window traps, and a Coleman lantern light trap (Muona 2000). In New Brunswick, this species was collected in Lindgren funnel traps in an old red oak forest with scattered white pine, in an old silver maple swamp, and in a red spruce forest with red maple and balsam fir. Adults were captured during July and August.

##### Distribution in Canada and Alaska.

ON, QC, **NB** (Bousquet 1991).

### Subfamily Eucneminae Eschscholtz, 1829

**Tribe Mesogenini Muona, 1993**

#### Stethon pectorosus

LeConte, 1866**

http://species-id.net/wiki/Stethon_pectorosus

Map 6

##### Material examined.

**New Brunswick, Queens Co.**, Grand Lake Meadows P.N.A., 45.8227°N, 66.1209°W, 19.VII-5.VIII.2011, M. Roy & V. Webster, old silver maple forest and seasonally flooded marsh, Lindgren funnel trap (1, RWC).

**Map 6.**
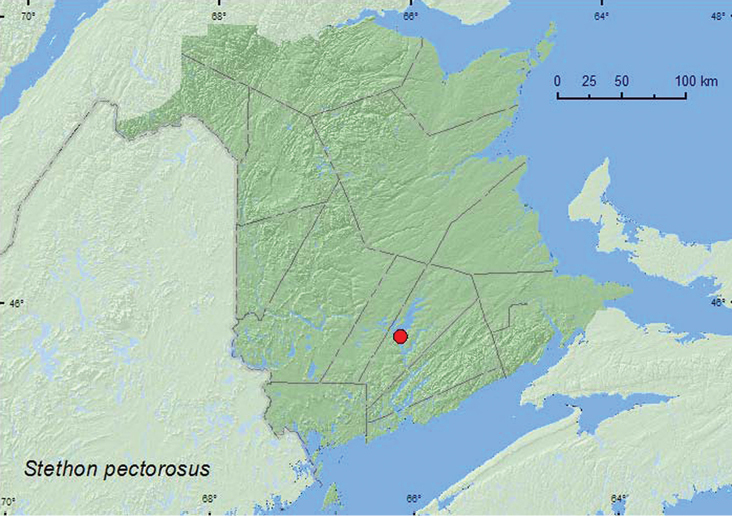
Collection localities in New Brunswick, Canada of *Stethon pectorosus*.

##### Collection and habitat data.

Most records of this species are from under bark of various species of deciduous trees, otherwise little is known about its biology (Muona 2000). The adult from New Brunswick was captured between late July and early August in a Lindgren funnel trap deployed in an old silver maple forest.

##### Distribution in Canada and Alaska.

ON, QC, **NB** (Bousquet 1991).

### Subfamily Macraulacinae Fleutiaux, 1923

**Tribe Macraulacini Fleutiaux, 1923**

#### Onichodon canadensis

(Brown, 1940)

http://species-id.net/wiki/Onichodon_canadensis

Map 7

##### Material examined. 

**Additional New Brunswick records. Carleton Co.**, Jackson Falls, Bell Forest, 46.2200°N, 67.7231°W, 19–28.VII.2008, R. P. Webster, mature hardwood forest, Lindgren funnel trap (1, RWC). **Charlotte Co.**, 10 km NW of New River Beach, 45.2110°N, 66.6170°W, 29.VI–16.VII.2010, 16–26.VII.2010, R. Webster & C. MacKay, old growth eastern white cedar forest, Lindgren funnel traps (2, AFC). **Northumberland Co.**, Priceville, 19.VII.1972, N. Carter, window trap (1 AFC). **Queens Co.**, Grand Lake near Scotchtown, 45.8762°N, 66.1816°W, 9.VII.2006, R. P. Webster, (red) oak and maple forest, m.v. light (1, RWC); Grand Lake Meadows P.N.A., 45.8227°N, 66.1209°W, 5–19.VII.2011, 19.VII–5.VIII.2011, 5–17.VIII.2011, M. Roy & V. Webster, old silver maple forest and seasonally flooded marsh, Lindgren funnel traps (6, NBM, RWC); Cranberry Lake P.N.A., 46.1125°N, 65.6075°W, 20.VII–4.VIII.2011, 4–18.VIII.2011, M. Roy & V. Webster, Lindgren funnel traps in forest canopy (2, NBM, RWC). **Sunbury Co.**, Acadia Research Forest, 45.9866°N, 66.3841°W, 21–29.VII.2009, R. Webster & M.-A. Giguère, red spruce forest with red maple and balsam fir, Lindgren funnel traps (2, AFC, RWC). **York Co.**, 15 km W of Tracy off Rt. 645, 45.6848°N, 66.8821°W, 20–29.VII.2009, 4–11.VIII.2010, R. Webster & M.-A. Giguère, old red pine forest, Lindgren funnel traps (3, AFC, RWC); same locality and habitat data but 30.VI-13.VII.2010, 13–27.VII.2010, 27.VII-10.VIII.2010, R. Webster, C. MacKay, & K. Burgess, Lindgren funnel traps (most in forest canopy) (19, AFC, NBM, RWC); 14 km WSW of Tracy, S of Rt. 645, 45.6741°N, 66.8661°W, 30.VI-13.VII.2010, R. Webster & C. MacKay, old mixed forest with red and white spruce, red and white pine, balsam fir, eastern white cedar, red maple, and *Populus* sp., Lindgren funnel traps (3, AFC).

**Map 7.**
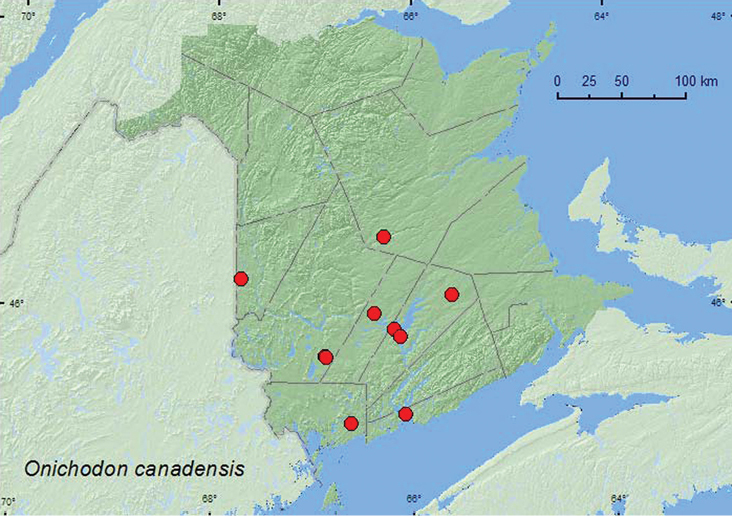
Collection localities in New Brunswick, Canada of *Onichodon canadensis*.

##### Collection and habitat data.

*Onichodon canadensis* has been reared from decayed yellow birch (*Betula alleghaniensis* Britt.), collected at black lights, and found on red spruce and *Fagus* sp. (Muona 2000). This species was found in various forest types in New Brunswick. These included a mature hardwood forest with beech, an old red oak and maple forest, an old red oak forest, an old silver maple swamp, an old mixed forest with red and white spruce (*Picea glauca* (Moench) Voss), red and white pine, balsam fir, eastern white cedar, red maple, and *Populus* sp., a red spruce forest with red maple and balsam fir, and an old-growth eastern white cedar forest. Most adults were captured in Lindgren funnel traps. One individual was collected at black-light trap, another in a window trap. Adults were captured during July and August.

##### Distribution in Canada and Alaska.

ON, QC, NB, PE, NS (Bousquet 1991; Majka 2007). Bousquet (1991) reported this species for New Brunswick, but no supporting voucher specimen was found in the CNC or other collections examined by Majka (2007). However, a specimen in the NBM collected by W. McIntosh in Saint John on 6 August 1900 was located, establishing this species as a member of the New Brunswick fauna (Majka 2007). The above records provide the first recent records of this species from the province.

#### Onichodon orchesides

Newman, 1838**

http://species-id.net/wiki/Onichodon_orchesides

Map 8

##### Material examined.

**New Brunswick, Carleton Co.**, Jackson Falls, Bell Forest, 46.2200°N, 67.7231°W, 12–19.VII.2008, 19–31.VII.2008, R. P. Webster, mature hardwood forest, Lindgren funnel traps (2, AFC, RWC); same locality and habitat but 19–31.VII.2009, R. Webster & M.-A. Giguère, Lindgren funnel trap (1, RWC). **Queens Co.**, Cranberry Lake P.N.A, 46.1125°N, 65.6075°W, 21–28.VII.2009, R. Webster & M.-A. Giguère, old red oak forest, Lindgren funnel trap (1, RWC); Grand Lake Meadows P.N.A., 45.8227°N, 66.1209°W, 19.VII–5.VIII.2011, M. Roy & V. Webster, old silver maple forest and seasonally flooded marsh, Lindgren funnel trap (1, RWC). **York Co.**, 15 km W of Tracy off Rt. 645, 45.6848°N, 66.8821°W, 13–27.VII.2010, R. Webster & C. MacKay old red pine forest, Lindgren funnel trap (1, RWC).

**Map 8.**
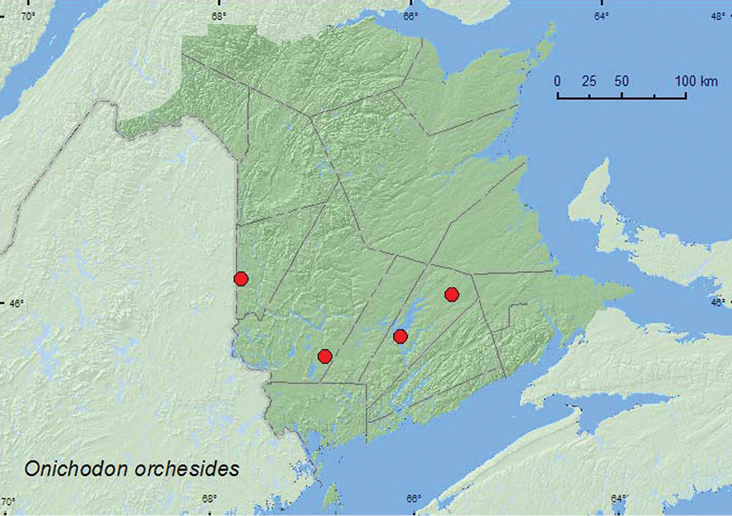
Collection localities in New Brunswick, Canada of *Onichodon orchesides*.

##### Collection and habitat data.

*Onichodon orchesides* has been collected in a light trap and from sugar maple, and remains of adults have been found in a rotten poplar log, otherwise little is known about its biology (Muona 2000). In New Brunswick, adults of this species were collected in a mature hardwood forest with American beech, an old red oak forest, an old silver maple forest, and an old red pine forest. All adults were captured in Lindgren funnel traps during July and August.

##### Distribution in Canada and Alaska.

ON, QC, **NB** (Bousquet 1991).

#### Isarthrus rufipes

(Melsheimer, 1844)**

http://species-id.net/wiki/Isarthrus_rufipes

Map 9

##### Material examined. 

**New Brunswick, Queens Co.**, Grand Lake Meadows P.N.A., 45.8227°N, 66.1209°W, 12–26.VII.2010, R. Webster & C. MacKay, old silver maple forest with green ash and seasonally flooded marsh, Lindgren funnel traps (2, RWC).

**Map 9.**
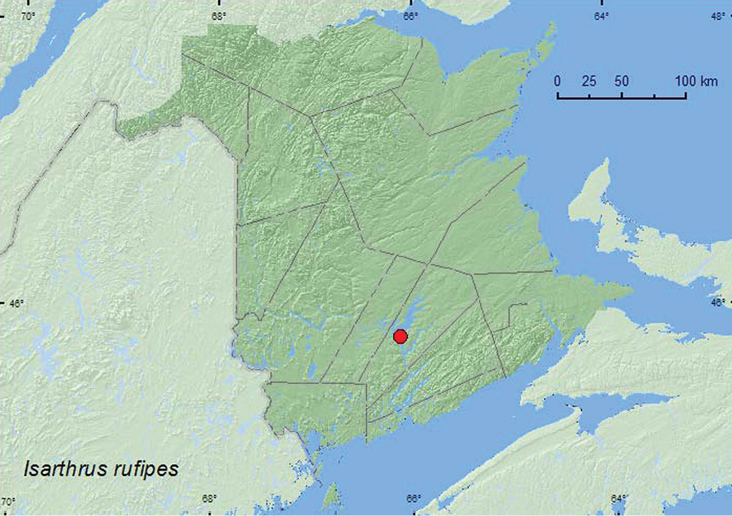
Collection localities in New Brunswick, Canada of *Isarthrus rufipes*.

##### Collection and habitat data.

This uncommon species has been reared from decayed American beech logs (Knull 1947) and rotten *Abies* sp., and collected in a malaise trap (Muona 2000). The two individuals from New Brunswick were caught in Lindgren funnel traps deployed in an old silver maple forest/swamp. Adults were captured during July.

##### Distribution in Canada and Alaska.

ON, **NB** (Muona 2000).

#### Dromaeolus harringtoni

Horn, 1886

http://species-id.net/wiki/Dromaeolus_harringtoni

Map 10

##### Material examined. 

**Additional New Brunswick Records. Queens Co.**, Cranberry Lake P.N.A, 46.1125°N, 65.6075°W, 15–21.VII.2009, 21–28.VII.2009, 28.VII–6.VIII.2009, 6–14.VIII.2009, R. Webster & M.-A. Giguère, old red oak forest, Lindgren funnel traps (11, AFC, RWC); same locality data and forest type, 20.VII–4.VIII.2011, 4–18.VIII.2011, M. Roy & V. Webster, Lindgren funnel traps (10, AFC, NBM, RWC). **Sunbury Co.**, Acadia Research Forest, 45.9866°N, 66.3841°W, 21–29.VII.2009, 4–11.VIII.2009, 11–18.VIII.2009, R. Webster & M.-A. Giguère, red spruce forest with red maple and balsam fir, Lindgren funnel traps (3, AFC, RWC). **York Co.**, 15 km W of Tracy off Rt. 645, 45.6848°N, 66.8821°W, 13–27.VII.2010, 27.VII–10.VIII.2010, R. Webster, C. MacKay, & C. Hughes, old red pine forest, Lindgren funnel traps (2, AFC, RWC); 14 km WSW of Tracy, S of Rt. 645, 45.6741°N, 66.8661°W, 13–27.VII.2010, R. Webster & C. MacKay, old mixed forest with red and white spruce, red and white pine, balsam fir, eastern white cedar, red maple, and *Populus* sp., Lindgren funnel traps (2, AFC).

**Map 10.**
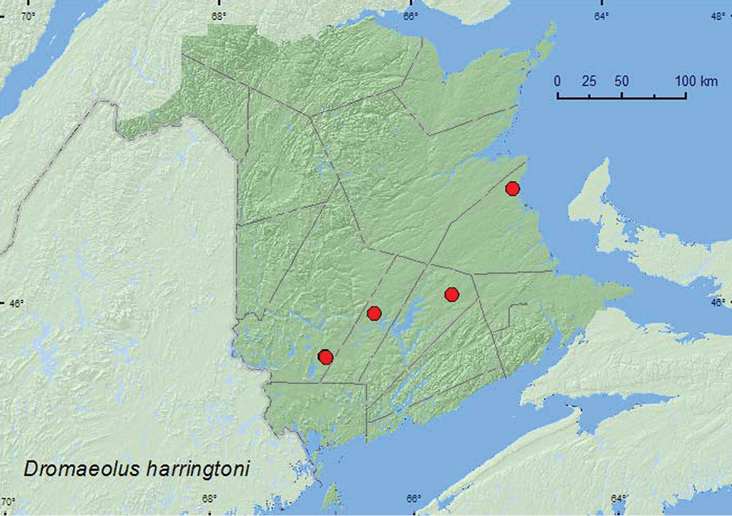
Collection localities in New Brunswick, Canada of *Dromaeolus harringtoni*.

##### Collection and habitat data.

*Dromaeolus harringtoni* was collected on beech (Dury 1888), otherwise little is known about the biology of this rare species (Muona 2000). In New Brunswick, this species (28 individuals) was captured in an old red oak forest, a red spruce forest with red maple and balsam fir, an old red pine forest, and an old mixed forest with red and white spruce, red and white pine (*Pinus strobus* L.), balsam fir, eastern white cedar, red maple, and *Populus* sp. All adults were captured in Lindgren funnel traps during July and August.

##### Distribution in Canada and Alaska.

MB, ON, QC, NB (Bousquet 1991). The first record of this species from New Brunswick was based on a specimen collected in the Kouchibouguac National Park (Kent Co.) by S.J. Miller (in CNC).

### Tribe Nematodini Leiler, 1976

#### Nematodes penetrans

(LeConte, 1852)

http://species-id.net/wiki/Nematodes_penetrans

Map 11

##### Material examined.

**New Brunswick, Queens Co.**, Grand Lake Meadows P.N.A., 45.8227°N, 66.1209°W, 29.VI-12.VII.2010, R. Webster, C. MacKay, M. Laity, & R. Johns, old silver maple forest with green ash and seasonally flooded marsh, Lindgren funnel trap (1, RWC); same locality data and forest type, 19.VII–5.VIII.2011, 5–17.VIII.2011, M. Roy & V. Webster, Lindgren funnel traps in forest canopy (5, AFC, NBM, RWC); Cranberry Lake P.N.A, 46.1125°N, 65.6075°W, M. Roy & V. Webster, 13–20.VII.2011, 20.VII–4.VIII.2011, 4–18.VIII.2011, old red oak forest, Lindgren funnel traps in forest canopy (22, AFC, NBM, RWC).

**Map 11.**
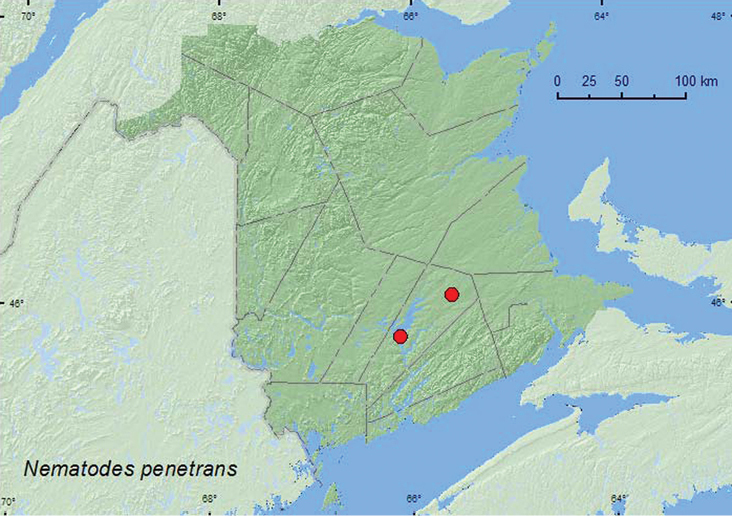
Collection localities in New Brunswick, Canada of *Nematodes penetrans*.

##### Collection and habitat data.

*Nematodes penetrans* was reared from American beech **(**Knull 1947) and from dead standing *Acer*, *Fagus*, and *Ulmus* spp. (Dury 1904). In New Brunswick, this species was captured during July and August in Lindgren funnel traps in an old silver maple forest with green ash and an old red oak forest. All but one individual were captured in traps deployed in the forest canopy.

##### Distribution in Canada and Alaska.

ON, QC, **NB**, NS (Bousquet 1991; Majka 2007).

## Discussion

Majka (2007) noted that several species of Eucnemidae have been infrequently collected in the Maritime provinces. *Microrhagus triangularis*, *Dromaeolus harringtoni*, and *Nematodes penetrans*, for example, were known from less than five specimens in the region. During our study, *Nematodes penetrans* (28 specimens), *Microrhagus triangularis* (six specimens), and *Dromaeolus harringtoni* (28 specimens) were captured in sizeable numbers in Lindgren funnel traps in New Brunswick (Table 1). Muona (2000) noted that *Dromaeolus harringtoni* appears to have declined in recent years in the United States, as there were no records after 1972. Otto (2010) collected it recently (2005) in several sites in Wisconsin employing flight-intercept traps and noted that “its decline may be related to the conversions of forest lands for agriculture and industrial uses, particularly in the late 19^th^ and early 20^th^ centuries.” In New Brunswick, this species was collected in Lindgren funnel traps at four of the six sites sampled using these traps, suggesting that this species is not uncommon here. Nine other eucnemid species were also captured in numbers of five or more individuals over the past 4 years in these traps. Indeed, six of the nine eucnemid species newly recorded for New Brunswick in this study were collected solely in Lindgren traps, and nearly 90% of all individuals of this family and all 15 species known from the province were captured in Lindgren funnel traps between 2008 and 2011, indicating that Lindgren funnel traps are effective for sampling members of this family.

## Supplementary Material

XML Treatment for Xylophilus cylindriformis

XML Treatment for Hylis terminalis

XML Treatment for Microrhagus subsinuatus

XML Treatment for Microrhagus triangularis

XML Treatment for Entomophthalmus rufiolus

XML Treatment for Stethon pectorosus

XML Treatment for Onichodon canadensis

XML Treatment for Onichodon orchesides

XML Treatment for Isarthrus rufipes

XML Treatment for Dromaeolus harringtoni

XML Treatment for Nematodes penetrans

## References

[B1] Blatchley WS (1910) An illustrated descriptive catalogue of the Coleoptera or beetles (exclusive of the Rhynchophora) known to occur in Indiana. The Nature Publishing Company, Indianapolis, Indiana, USA, 1386 pp. doi: 10.5962/bhl.title.56580

[B2] Bouchard P Bousquet Y Davies AE Alonso-Zarazaga MA Lawrence JF Lyal CHC Newton AF Reid CAM Schmitt M Ślipiński SA Smith ABT (2011) Family-group names in Coleoptera (Insecta). ZooKeys 88: 1-972. doi: 10.3897/zookeys.88.807 PMC3088472 21594053

[B3] Bousquet Y (1991) Family Eucnemidae: false click beetles. [pp. 186–188] In: Bousquet Y (Ed) Checklist of Beetles of Canada and Alaska. Agriculture Canada, Ottawa, Ontario, Publication 1861/E, 430 pp.

[B4] Dury C (1888) Elateridae in the vicinity of Cincinnati, Ohio. Entomologica Americana 4: 163-164.

[B5] Dury C (1904) Notes on Coleoptera. Entomological News 15: 52-53.

[B6] Horn GH (1886) A monograph of the species of the subfamilies Eucneminae, Cerophytinae and Perothopinae inhabiting the United States. Transactions of the American Entomological Society 20: 5-58.

[B7] Knull JM (1946) A new species of *Dirhagus* with notes on other Eucnemidae (Coleoptera). Annals of the Entomological Society of America 39: 246-247.

[B8] Knull JM (1947) New Elateridae with notes on Eucnemidae. Entomological News 58: 177-181.

[B9] Levesque C Levesque G-Y (1993) Abundance and seasonal activity of Elateroidea (Coleoptera) in a raspberry plantation and adjacent sites in southern Quebec, Canada. The Coleopterists Bulletin 47: 269-277.

[B10] Lindgren BS (1983) A multiple funnel trap for scolytid beetles (Coleoptera). The Canadian Entomologist 115: 299-302. doi: 10.4039/Ent115299-3

[B11] Majka CG (2007) The Eucnemidae (Coleoptera) of the Maritime provinces of Canada: new records, observations on composition and zoogeography, and comments on the rarity of saproxylic beetles. Zootaxa 1636: 33-46.

[B12] Muona J (1993) Review of the phylogeny, classification and biology of the family Eucnemidae (Coleoptera). Entomologica Scandinavica Supplement 44, 133 pp.

[B13] Muona J (2000) Revision of the Nearctic Eucnemidae. Acta Zoologica Fennica 212: 1-106.

[B14] Otto RL (2010) New records for seven rare Nearctic species of false click beetles (Coleoptera: Eucnemidae). The Coleopterists Bulletin 64: 92-93. doi: 10.1649/0010-065X-64.1.92

[B15] Webster RP Klimaszewski J Pelletier G Savard K (2009) New Staphylinidae (Coleoptera) records with new collection data from New Brunswick, Canada. I. Aleocharinae. In: Majka CG Klimaszewski J (Eds). Biodiversity, biosystematics, and ecology of Canadian Coleoptera II. ZooKeys 22: 171–248. doi: 10.3897/zookeys.22.152

[B16] Webster RP Smetana A Sweeney JD DeMerchant I (in press) New Staphylinidae (Coleoptera) records with new collection data from New Brunswick and an addition to the fauna of Quebec: Staphylininae. In: Klimaszewski J, Anderson R (Eds) Biodiversity, Biosystematics and Ecology of Canadian Staphylinidae (Coleoptera) II. ZooKeys. 10.3897/zookeys.186.2469 PMC3349199 22577325

